# Effects of central apneas on sympathovagal balance and hemodynamics at night: impact of underlying systolic heart failure

**DOI:** 10.1007/s11325-020-02144-8

**Published:** 2020-07-22

**Authors:** Jens Spiesshoefer, Nora Hegerfeld, Malte Frank Gerdes, Sören Klemm, Martha Gorbachevski, Robert Radke, Izabela Tuleta, Claudio Passino, Xiaoyi Jiang, Paolo Sciarrone, Winfried Randerath, Michael Dreher, Matthias Boentert, Alberto Giannoni

**Affiliations:** 1 Institute of Life Sciences, Scuola Superiore Sant’Anna, Piazza Martiri della Libertà, 33, 56127 Pisa, PI Italy; 2 Department of Neurology with Institute for Translational Neurology, University of Muenster, Muenster, Germany; 3 Faculty of Mathematics and Computer Science, University Muenster, Muenster, Germany; 4 Department of Cardiology III, University Hospital Muenster, Muenster, Germany; 5 Department of Cardiology I, University Hospital Muenster, Muenster, Germany; 6 Cardiology and Cardiovascular Medicine Division, Fondazione Toscana Gabriele Monasterio, National Research Council, CNR-Regione Toscana, Pisa, Italy; 7 Bethanien Hospital gGmbH, Solingen, Germany; 8 Institute for Pneumology at the University of Cologne, Solingen, Germany; 9 Department of Pneumology and Intensive Care Medicine, University Hospital RWTH, Aachen, Germany; 10 Department of Medicine, UKM Marienhospital, Steinfurt, Germany

**Keywords:** Central apnea, Heart failure, Sympathovagal balance

## Abstract

**Background:**

Increased sympathetic drive is the key determinant of systolic heart failure progression, being associated with worse functional status, arrhythmias, and increased mortality. Central sleep apnea is highly prevalent in systolic heart failure, and its effects on sympathovagal balance (SVB) and hemodynamics might depend on relative phase duration and background pathophysiology.

**Objective:**

This study compared the effects of central apneas in patients with and without systolic heart failure on SVB and hemodynamics during sleep.

**Methods:**

During polysomnography, measures of SVB (heart rate and diastolic blood pressure variability) were non-invasively recorded and analyzed along with baroreceptor reflex sensitivity and hemodynamic parameters (stroke volume index, cardiac index, total peripheral resistance index). Data analysis focused on stable non-rapid eye movement N2 sleep, comparing normal breathing with central sleep apnea in subjects with and without systolic heart failure.

**Results:**

Ten patients were enrolled per group. In heart failure patients, central apneas had neutral effects on SVB (all *p* > 0.05 for the high, low, and very low frequency components of heart rate and diastolic blood pressure variability). Patients without heart failure showed an increase in very low and low frequency components of diastolic blood pressure variability in response to central apneas (63 ± 18 vs. 39 ± 9%; *p* = 0.001, 43 ± 12 vs. 31 ± 15%; *p* = 0.002). In all patients, central apneas had neutral hemodynamic effects when analyzed over a period of 10 min, but had significant acute hemodynamic effects.

**Conclusion:**

Effects of central apneas on SVB during sleep depend on underlying systolic heart failure, with neutral effects in heart failure and increased sympathetic drive in idiopathic central apneas.

**Electronic supplementary material:**

The online version of this article (10.1007/s11325-020-02144-8) contains supplementary material, which is available to authorized users.

## Introduction

Patients with systolic heart failure (HF) often present with an unstable respiratory pattern characterized by alternating phases of hyperventilation and central apneas (CA), also named Cheyne-Stokes respiration (CSR) [1–3]. CSR is mainly caused by increased chemosensitivity to either hypoxia or hypercapnia [4] and delayed chemoresponse due to the prolonged circulatory time that directly results from decreased cardiac output [5]. While the former seems to influence CSR severity, the latter seems to rather influence CSR cycle duration, and the duration of the hyperventilation phase in particular [5]. It has been hypothesized that CSR alters sympathovagal balance (SVB) in favor of increased sympathetic drive and thus has detrimental effects in HF when left untreated [3, 6]. This may be mainly due to an altered pulmonary stretch reflex and blunted vagal stimulation [6, 7], but also due to chemoreflex-mediated sympathetic overactivity in response to apnea-related hypoxemia and hypercapnia [7, 8]. SVB is known to play a key role in determining HF progression because medications that decrease sympathetic drive have been shown to have favorable effects on outcome [9, 10]. For this reason, CA has been considered a potential therapeutic target in HF and has been treated using a variety of options, including oxygen therapy [11], carbon dioxide (CO_2_) rebreathing [11, 12], acetazolamide [13–15], and nocturnal mask-based therapies such as continuous positive airway pressure [16] or adaptive servo-ventilation [17, 18].

The results of the Treatment of Sleep-Disordered Breathing with Predominant Central Sleep Apnea by Adaptive Servo Ventilation in Patients with Heart Failure (SERVE-HF trial) [19] have challenged this hypothesis, because patients with systolic HF (left ventricular ejection fraction [LVEF] ≤ 45%) and CSR treated with adaptive servo-ventilation unexpectedly showed increased mortality compared with controls who received optimal medical therapy alone [20, 21]. This finding has led to controversy about the effect of CSR in HF as potentially compensatory rather than harmful, at least in a subset of patients [20, 21]. Potential favorable effects of CSR are thought to include an increase in venous return and cardiac output, and positive effects of hypocapnia on SVB during the hyperventilation phase [22, 23].

The net effect of CSR, which is characterized by alternating cycles of hyperventilation and CA may be influenced by several factors. These include the relative duration of the hyperventilation and CA phases, as well as background cardiac hemodynamics and/or feedback resetting. CA is also seen, albeit less frequently, in subjects in whom no overt cardiac or neurological cause can be identified, where it is called idiopathic central apnea (ICA) [24]. In patients with ICA, apneic events are usually associated with increased sensitivity of the chemoreflex and are characterized by shorter cycle duration resulting from normal cardiac output and circulation time [25]. ICA may also potentially alter SVB, either directly or indirectly (by altering sleep quality), but this (along with its potential hemodynamic effects) has not yet been investigated.

This study investigated the immediate effects of CA on SVB and hemodynamic parameters during stage 2 non-rapid eye movement (NREM) sleep (N2) in patients with HF-related CA and ICA. This approach allows evaluation of whether the effects of CA on both the autonomous nervous system and the cardiovascular system during sleep differ between patients with and without systolic HF. Thus, it would theoretically be possible to identify a mechanism by which CA potentially exerts beneficial effects in HF patients.

## Materials and methods

### Study design and study population

Patients with CA (apnea-hypopnea index [AHI] ≥ 15/h with > 50% central events and a central AHI of ≥ 10/h [i.e., SERVE-HF-like inclusion criteria]) due to systolic HF (LVEF < 50% [19], N-terminal pro B-type natriuretic peptide [NT-proBNP] level ≥ 125 ng/L, but by inclusion criterion in sinus rhythm to have valid data on HRV and dBPV) and heart-healthy subjects with ICA (same CA criteria as HF patients, but with LVEF ≥ 55%, sinus rhythm, and serum NT-proBNP < 125 ng/L) from the University Hospital of Muenster were consecutively recruited from April 2018 to February 2019. Diagnosis of CA had previously been established by diagnostic polysomnography (PSG). The study protocol conforms to the 1975 Declaration of Helsinki and was approved by the Institution’s human research committee (Ethikkommission der Ärztekammer Westfalen-Lippe und der Medizinischen Fakultät der Westfälischen Wilhelms-Universität Münster, AZ. 2017-188-f-S). All participants gave written informed consent to participate in the study, and the project had been registered under the German Clinical Trials Registry (drks.de identifier: DRKS00013883).

### Assessments

All study participants underwent standard 2-dimensional Doppler echocardiography (LOGIQ S8-XD clear, GE Healthcare, London, UK), standard lung function tests (Vitalograph ® 3000, Vitalograph, Hamburg, Germany) performed according to current recommendations, and measurement of serum NT-proBNP [26]. Furthermore, study participants underwent non-invasive hemodynamic and respiratory monitoring during sleep, as detailed below.

### Non-invasive hemodynamic and respiratory monitoring

**Fig. 1:**
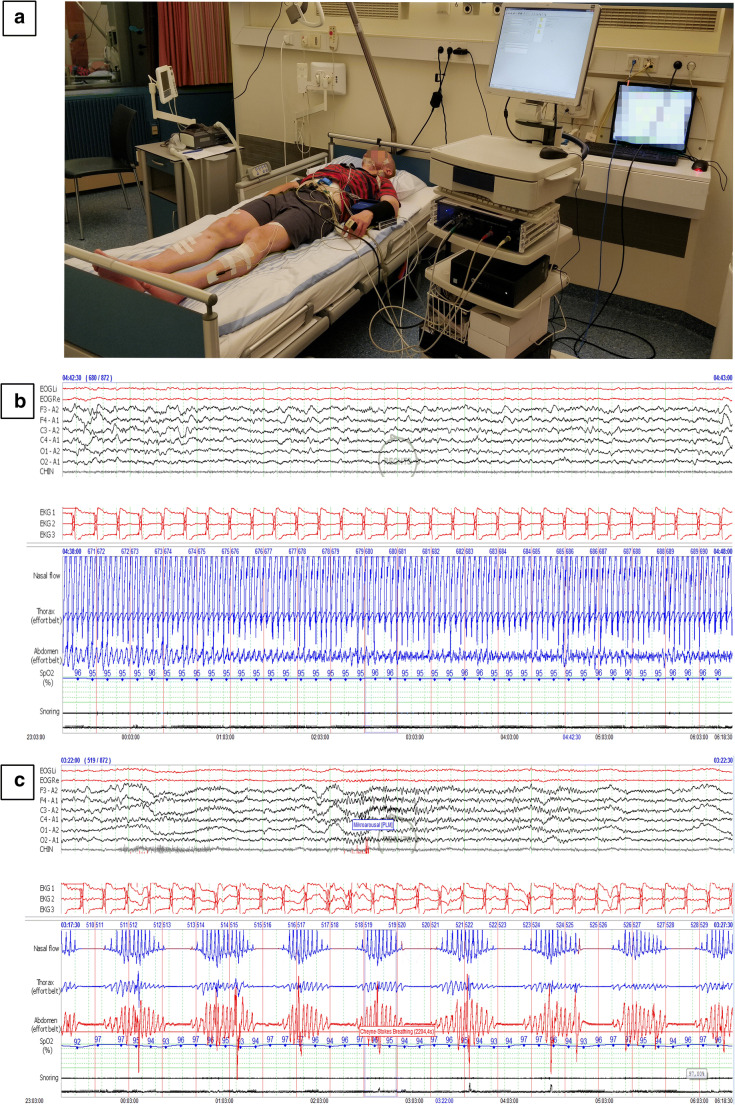
Overview of the experimental setup—a sample patient being connected with the respiratory, polysomnography, and non-invasive autonomic nervous system function monitoring systems (**a**), and representative respiratory and polysomnography readings taken from stable deep sleep contrasting a normal breathing pattern (**b**), and a central apnea-like breathing pattern (**c**)

Parameters reflecting SVB and hemodynamic parameters were measured during sleep using a non-invasive monitor device (Task Force Monitor®, CNSystems, Graz, Austria) as previously validated (Fig. 1a) [27–29]. For the assessment of SVB, heart rate variability (HRV) and diastolic blood pressure variability (dBPV) were analyzed using a 3-lead electrocardiogram (sampling rate 1000 Hz) and a continuous non-invasive arterial blood pressure signal (CNAP® technology) with a sampling rate of 100 Hz. HRV and dBPV were computed by frequency domain analysis of continuous recordings of RR intervals (for HRV) or blood pressure changes (for dBPV). HRV (expressed as ms^2^) and dBPV (expressed as mmHg^2^) were presented as the high frequency component (HF; 0.15–0.4 Hz), low frequency component (LF; 0.04–0.15 Hz), their relative ratio (LF/HF), and the very low frequency component (VLF; 0.0–0.04 Hz) [27, 30, 31]. Given that total power spectra of HRV and dBPV are significantly reduced in patients with HF compared with heart-healthy individuals, LF, HF, and VLF data for both HRV and dBPV were reported as normalized unit (n.u.) for total power spectra [32].

Baroreceptor reflex sensitivity (BRS) was measured using the sequence method [33, 34].

Non-invasive hemodynamic measurements included beat-to-beat systolic (sBP) and diastolic (dBP) blood pressure; these were validated against periodic measurements obtained every 60 min by oscillometric recording from the upper contralateral arm. Transthoracic impedance measurements were used to estimate cardiac stroke volume index (SVI), cardiac index (CI), and systemic vascular resistance (SVR), from which the total peripheral resistance index (TPRI) was calculated (mean blood pressure divided by CI). Bioimpedance-based measurements have been previously validated against invasive hemodynamic monitoring [27–29]. All signals were simultaneously acquired and displayed in real time on a personal computer running DOMINO 2.9.0 software.

Respiration was monitored using a polysomnograph device (SOMNO HD™, SOMNOmedics GmbH, Randersacker, Germany), recording airflow through a nasal cannula thermistor (TerniMed, Bielefeld, Germany), thoracic and abdominal efforts through inductance belts, and an electroencephalogram (EEG) through a 12-lead EEG system, according to recommendations of the American Academy of Sleep Medicine (AASM) [35]. Sleep stages and respiratory events were also analyzed based on AASM recommendations [35].

### Selection of central apnea and normal breathing segments

Participants were connected to non-invasive hemodynamic and respiratory monitoring systems and spent the following night in the academic sleep laboratory at Münster University Hospital. Figure 1a shows a patient in a sleep lab bed being connected to the non-invasive hemodynamic and respiratory monitoring systems. Sleep stages, respiration, hemodynamic, and autonomic nervous system parameters were evaluated online and on a “beat-to-beat” basis by NH and JS.

From each subject, three fragments were chosen by visual identification, each of them 10 min in duration. The first segment was characterized by normal breathing (NB) during the awake state, the second by NB during stable N2 sleep, and the third segment by CA during stable N2 sleep. With regard to HRV analysis, all segments selected were required to show sinus rhythm with fewer than 5% ectopic beats. SVB and BRS were computed in the same time windows of 10 min.

Normal breathing was defined as the absence of apneas, hypopneas, and respiratory event-related arousals from sleep (RERA). Variability of peripheral oxygen saturation (SpO_2_) was required to be < 2% over > 80% of the time during the 10-min segment. Selection of 10-min segments of N2 sleep with CA was based on the presence of CAs for ≥ 20% of the time. A CA was scored when flow amplitude was decreased by ≥ 90% for ≥ 10 s in the absence of any ventilatory effort. CA were visually identified and manually selected for in-depth analysis of associated changes in hemodynamic measures.

Average values for HR, sBP, and dBP were calculated during the CA and compared with the 15 s preceding the apnea. The 15 s preceding the apnea had to be characterized by NB, as defined above. Representative tracings of a CA breathing pattern and NB during stable N2 sleep are shown Fig. 1b and c.

### Statistical analyses

All analyses were performed using the Sigma Plot® software (Version 13.0, Systat Software GmbH, Erkrath, Germany). Assuming a two-sided significance level of 0.05 (alpha) and 80% power (beta), a sample size of ten patients per group was calculated to allow detection of a 20% change in the LF component of dBPV. Values for the mean and standard deviation of LF component of dBPV during 10 min of N2 sleep were obtained from previous preliminary data [36, 37]. Furthermore, it was also known from our previous measurements that intra-individual variation in the LF component of dBPV during N2 sleep is ~ 5% at most [37]. Results were expressed as mean and standard deviation for continuous variables with normal distribution, and median and interquartile range for continuous variables with a skewed distribution. Categorical variables were expressed as percentages, unless otherwise specified. Differences between groups (HF and ICA patients) were analyzed using the unpaired *t* test or the Mann-Whitney rank sum test, when appropriate, while differences in categorical data were compared using the *χ*^2^ test. Measures reflecting SVB and hemodynamic parameters were compared with baseline values using a paired *t* test or Wilcoxon rank sum test, as appropriate. For all tests, a two-tailed *p* value ≤ 0.05 was considered statistically significant.

## Results

### Subjects

A total of 20 subjects were enrolled, 10 with CA due to HF and 10 with ICA (Table 1). Patients with HF had lower LVEF and higher plasma levels of NT-proBNP, consistent with the inclusion criteria. Etiology of HF was ischemic in 6/10 patients. There were no significant differences in age, gender, body mass index, and basic lung function or basic objective sleep data of the study night (Supplemental Table S2) between the two patient groups. The majority of patients with systolic HF were taking a β-blocker and angiotensin-converting enzyme (ACE) inhibitor, as recommended by current guidelines [10]. Etiology of HF was ischemic in 6/10 patients. Among patients with ICA, 4/10 were taking a β-blocker and/or ACE inhibitor for pharmacological treatment of arterial hypertension. Supplemental Table 1 reports the overall sleep-related characteristics of the study night.

**Table 1:** Demographic and clinical characteristics of the study population at baseline

	CA-HF (*n* = 10)	ICA (*n* = 10)	*p* value
Male (*n* (%))	9 (90)	9 (90)	1.0
Age (years)	65.2 ± 10.8	57.7 ± 13.2	0.22
BMI (kg/m^2^)	29.1 ± 4.6	29.6 ± 5.3	0.84
BSA (m^2^)	2.0 ± 0.2	2.1 ± 0.2	0.47
NYHA class I (*n* (%))	0 (0)	n.a.	
NYHA class II (*n* (%))	7 (70)	n.a.	
NYHA class III (*n* (%))	3 (30)	n.a.	
NYHA class IV (*n* (%))	0 (0)	n.a.	
LVEF (%)	36.6 ± 11.8	63.3 ± 5.5	*< 0.001*
Mitral regurgitation (grade III) (*n* (%))	0 (0)	0 (0)	1.0
TAPSE (mm)	19.7 ± 4.5	25.1 ± 3.1	*0.008*
NT-proBNP (ng/L)	962.5 (799.5–1738.0)	60.5 (50.0–113.0)	*< 0.001*
Lung function data			
FVC (L)	3.6 ± 1.1	4.5 ± 1.4	0.16
FVC (% predicted)	93.7 ± 24.2	104.2 ± 29.8	0.41
FEV_1_ (L)	3.0 ± 0.7	3.6 ± 1.0	0.15
FEV_1_ (% predicted)	96.4 ± 19.2	103.9 ± 25.1	0.48
FEV_1_/VC (%)	76.0 ± 7.5	70.5 ± 7.1	0.12
PEF (L/s)	418.9 ± 125.4	442.3 ± 159.6	0.73
Medication (*n* (%))			
ACEI/ARB	9 (90)	4 (40)	*0.018*
β-Blocker	10 (100)	3 (30)	*< 0.001*
Diuretics	8 (80)	1 (10)	*< 0.001*
Aldosterone antagonists	6 (60)	-	*0.002*

Italicized means *p* < 0.05

Values are mean ± standard deviation, median (interquartile range), or number of patients (%)

*ACEI*, angiotensin-converting enzyme inhibitor; *ARB*, angiotensin receptor blocker; *BMI*, body mass index; *BSA*, body surface area; *CA-HF*, central apnea and heart failure patients; *FEV*_*1*_, forced expiratory volume; *FVC*, forced vital capacity; *ICA*, idiopathic central sleep apnea patients; *LVEF*, left ventricular ejection fraction; *NT-proBNP*, N-terminal pro brain natriuretic peptide; *NYHA*, New York Heart Association; *PEF*, peak expiratory flow; *TAPSE*, tricuspid annular plane systolic excursion; *VC*, vital capacity

### Baseline sympathovagal balance and hemodynamics during free breathing

Measures reflecting SVB, BRS, and hemodynamic parameters during free breathing are detailed in Table 2. Patients with HF and CA showed a reduced BRS (slope < 15 ms/mmHg) in 40% and 50% of cases considering up events and down events, respectively. Subjects with ICA showed reduced BRS in 90% and 70% of cases again considering up events and down events, respectively. [38]. HF patients had lower blood pressure, SVI, and CI compared with ICA patients (all *p* < 0.01). There were no significant differences in HR or total peripheral resistance index (TPRI) between HF and ICA patients (all *p* > 0.05).

**Table 2:** Sympathovagal balance and hemodynamics in patients with heart failure-related central apnea and patients with idiopathic central apnea during free breathing in the awake state

	CA-HF	ICA	*p* value
Sympathovagal balance parameters			
HF nuRRI (%)	71.2 ± 15.0	39.9 ± 29.5	*0.01*
LF nuRRI (%)	28.8 ± 15.0	60.1 ± 29.5	*0.01*
LF/HF RRI	0.5 ± 0.4	2.8 ± 2.3	*0.006*
HF nudBPV (%)	30.1 ± 12.0	22.5 ± 20.6	0.34
LF nudBPV (%)	30.5 ± 8.6	37.6 ± 14.0	0.20
LF/HF dBPV	1.1 ± 0.4	4.1 ± 5.0	0.07
VLF nudBP (%)	39.4 ± 18.2	39.9 ± 17.2	0.95
BRS slope			
Up events (*n*)	52	91	0.16
Up events (ms/mmHg)	33.9 ± 28.6	11.5 ± 9.9	0.07
BR slope < 15 ms/mmHg (*n* (%))	40	90	*0.04*
Down events (*n*)	58	105	*0.04*
Down events (ms/mmHg)	36.1 ± 30.3	14.1 ± 11.0	0.07
% with BRS slope < 15 ms/mmHg (*n* (%))	50	70	0.096
Hemodynamic parameters			
Heart rate (min^−1^)	68.5 ± 8.3	62.4 ± 8.8	0.15
Systolic BP (mmHg)	98.3 ± 25.1	104.9 ± 11.5	0.51
Diastolic BP (mmHg)	59.8 ± 23.5	63.3 ± 13.0	0.71
Stroke volume index (mL/m^2^)	26.3 ± 5.4	35.0 ± 4.9	*0.003*
Cardiac index (L/min/m^2^)	1.8 ± 0.4	2.2 ± 0.4	*0.05*
TPRI (dyne s m^2^ cm^−5^)	3608.9 ± 1482.3	2997.9 ± 511.7	0.28

Italicized means *p* < 0.05

Values are mean ± standard deviation or number of patients (%)

*BP*, blood pressure; *BRS slope*, slope of baroreceptor reflex sensitivity (up events and down events); *CA-HF*, central apnea and heart failure patients; *HF nudBPV*, high frequency component of diastolic blood pressure variability normalized for total power spectra (normalized units); *HF nuRRI*, high frequency component of heart rate variability normalized for total power spectra (normalized units); *ICA*, idiopathic central sleep apnea patients; *LF nudBPV*, low frequency component of diastolic blood pressure variability normalized for total power spectra (normalized units); *LF/HF dBPV*, relative ratio of low frequency and high frequency component of diastolic blood pressure variability; *LF nuRRI*, low frequency component of heart rate variability normalized for total power spectra (normalized units); *LF/HF RRI*, relative ratio of low frequency and high frequency component of heart rate variability; *TPRI*, total peripheral resistance index

### Impact of CA on sympathovagal balance and hemodynamics in patients with HF

In HF patients, transition from the awake state to N2 sleep under NB was not associated with statistically significant changes in any SVB parameters, but there were significant reductions from baseline to sleep in hemodynamic variables with a reduction in heart rate (by ~ 8%, *p* = 0.009), SVI (by ~ 7%, *p* = 0.02), and CI (by ~ 15%, *p* = 0.002) (Table 3).

**Table 3:** Effect of central apnea on sympathovagal balance and hemodynamic parameters in patients with heart failure

	Baseline awake (NB)	N2 sleep (NB)	*p* value*	N2 sleep (CA)	*p* value**
Sympathovagal balance parameters					
HF nuRRI (%)	71.2 ± 15.0	65.3 ± 19.0	0.50	62.3 ± 24.0	0.28
LF nuRRI (%)	28.8 ± 15.0	34.7 ± 19.0	0.50	37.7 ± 24.0	0.28
LF/HF RRI	0.5 ± 0.4	0.8 ± 0.8	0.35	1.1 ± 1.3	0.19
HF nudBPV (%)	30.1 ± 12.0	28.6 ± 13.2	0.61	27.6 ± 12.9	0.65
LF nudBPV (%)	30.5 ± 8.6	27.4 ± 10.3	0.19	27.0 ± 7.6	0.86
LF/HF dBPV	1.1 ± 0.4	1.1 ± 0.3	0.54	1.1 ± 0.3	0.73
VLF nudBP (%)	39.4 ± 18.2	44.0 ± 22.4	0.97	45.4 ± 18.9	0.75
BRS slope					
Up events (ms/mmHg)	33.9 ± 28.6	41.8 ± 31.4	0.66	41.7 ± 32.7	0.96
Down events (ms/mmHg)	36.1 ± 30.3	55.8 ± 38.6	0.14	36.0 ± 31.7	0.27
Hemodynamic parameters					
Heart rate (min^−1^)	68.5 ± 8.3	63.0 ± 10.2	*0.009*	64.1 ± 8.7	0.42
Systolic BP (mmHg)	98.3 ± 25.1	100.1 ± 23.8	0.81	94.8 ± 19.0	0.38
Diastolic BP (mmHg)	59.8 ± 23.5	63.8 ± 23.9	0.60	58.4 ± 17.9	0.41
Stroke volume index (mL/m^2^)	26.3 ± 5.4	24.3 ± 5.3	*0.02*	24.9 ± 5.0	0.47
Cardiac index (L/min/m^2^)	1.8 ± 0.4	1.5 ± 0.3	*0.002*	1.6 ± 0.3	0.19
TPRI (dyne s m^2^ cm^−5^)	3608.9 ± 1482.3	4053.4 ± 1067.7	0.13	3844.0 ± 1435.7	0.09

Italicized means *p* < 0.05

Values are mean ± standard deviation or number of patients (%)

*For comparison vs. baseline

**For comparison CA vs. NB

*BP*, blood pressure; *BRS slope*, slope of baroreceptor reflex sensitivity (up events and down events); *CA*, central apnea; *HF nudBPV*, high frequency component of diastolic blood pressure variability normalized for total power spectra (normalized units); *HF nuRRI*, high frequency component of heart rate variability normalized for total power spectra (normalized units); *LF nudBPV*, low frequency component of diastolic blood pressure variability normalized for total power spectra (normalized units); *LF/HF dBPV*, relative ratio of low frequency and high frequency component of diastolic blood pressure variability; *LF nuRRI*, low frequency component of heart rate variability normalized for total power spectra (normalized units); *LF/HF RRI*, relative ratio of low frequency and high frequency component of heart rate variability; *NB*, normal breathing; *TPRI*, total peripheral resistance index

**Fig. 2:**
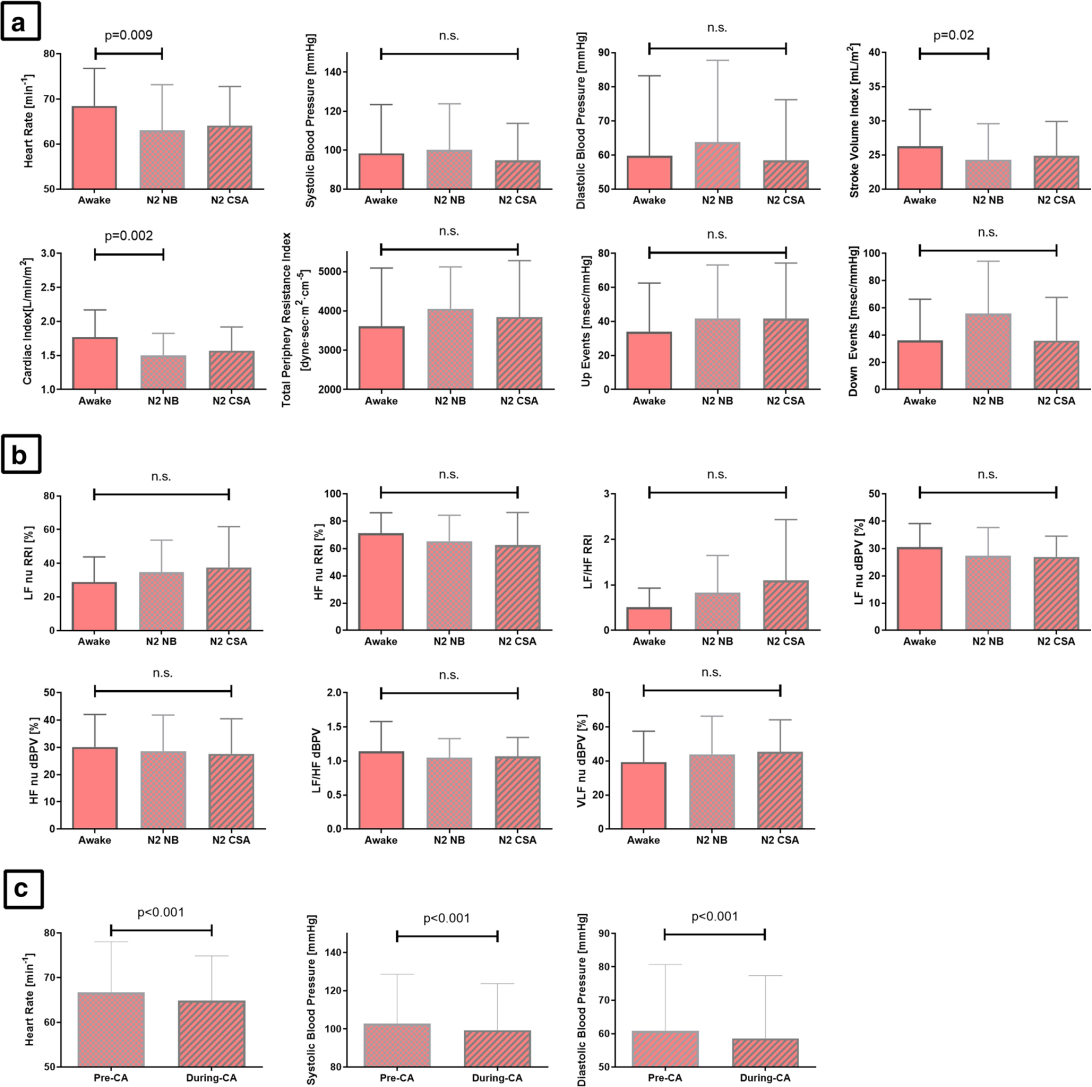
Changes in hemodynamic parameters (**a**) and sympathovagal balance parameters (**b**) comparing normal breathing (NB) and central sleep apnea (CA) in HF patients (there were no statistically significant changes in these parameters). Immediate effects of CAs on hemodynamics (**c**) were seen, such that heart rate (HR), systolic blood pressure (sBP), and diastolic blood pressure (dBP) all decreased during CAs compared with the pre-apneic phase. CSA, central sleep apnea; HF RRI, high frequency component of heart rate variability; LF RRI, low frequency component of heart rate variability; LF/HF RRI, relative ratio of low frequency and high frequency component of heart rate variability; N2, stage 2 non-rapid eye movement sleep

During stable N2 sleep, the phase of unstable breathing (25 ± 5% of the 10-min time segments chosen, i.e., corresponding to mild-moderate CA severity) was not associated with any significant variations in SVB and hemodynamics compared with the phase of NB (Table 3, Fig. 2 for a summary of the effects seen in the entire group of HF patients). When comparing hyperventilation and CA within the unstable breathing phase, there was a ~ 5% decrease in HR (*p* < 0.001), sBP (*p* < 0.001), and dBP (*p* < 0.001) during CA (*n* = 1233; 23 ± 5 s in length) compared with the last 15 s of hyperventilation preceding apnea onset (Fig. 2c).

### Impact of CA on sympathovagal balance and hemodynamics in patients with ICA

**Fig. 3:**
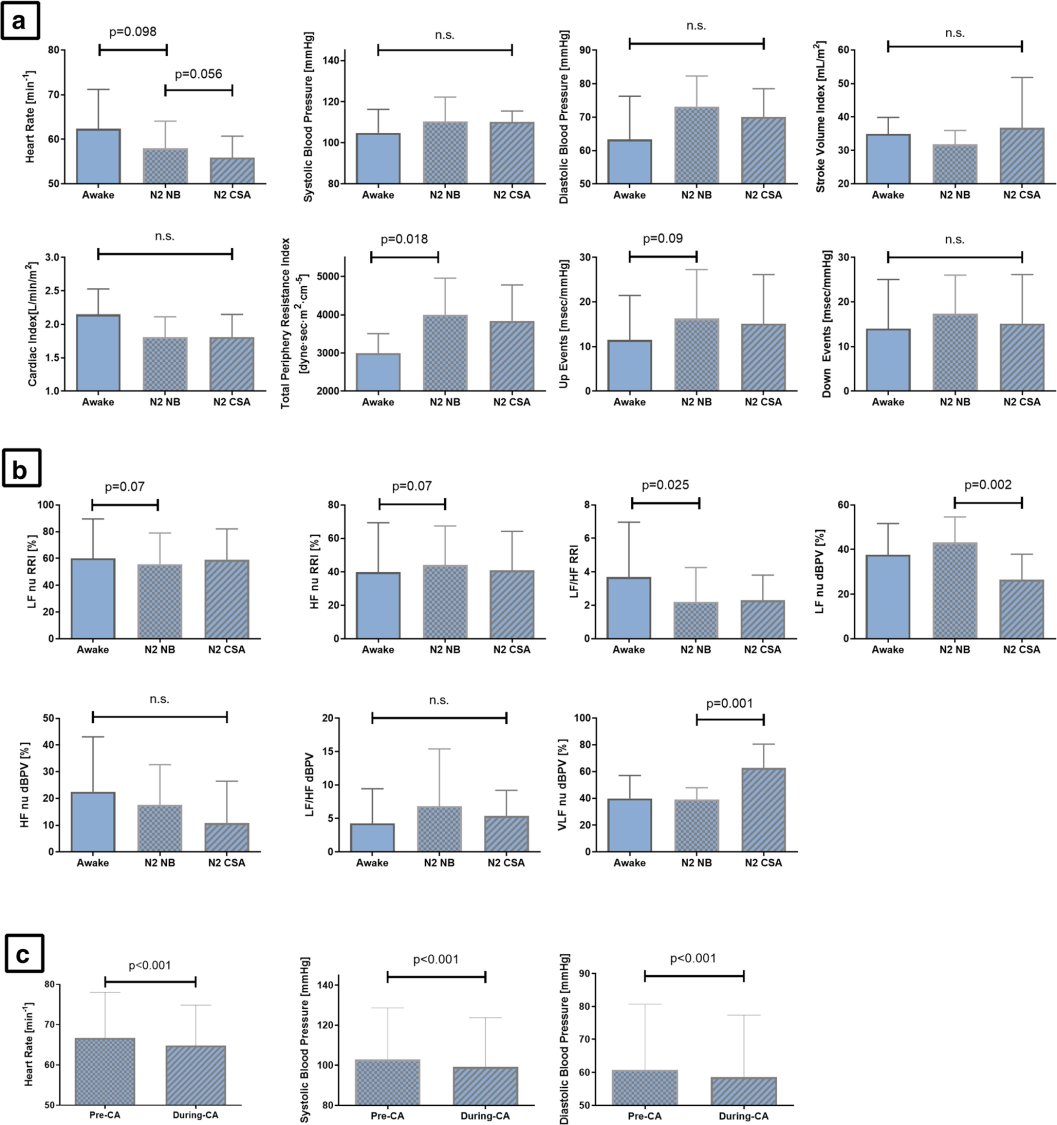
Changes in hemodynamic parameters (**a**) and sympathovagal balance parameters (**b**) comparing normal breathing (NB) and central apnea (CA) in idiopathic CA (ICA) patients; there was a significant increase in very low frequency components of diastolic blood pressure variability (VLF dBP) (*p* = 0.01). Immediate effects of CAs on hemodynamics (**c**) were seen, such that heart rate (HR), systolic blood pressure (sBP), and diastolic blood pressure (dBP) all decreased during CAs compared with the pre-apneic phase. CSA, central sleep apnea; HF RRI, high frequency component of heart rate variability; LF RRI, low frequency component of heart rate variability; LF/HF RRI, relative ratio of low frequency and high frequency component of heart rate variability; N2, stage 2 non-rapid eye movement sleep

In ICA patients, transition from the awake state to N2 sleep was associated with a reduction in LF/HF RRI (by ~ 40%) under NB conditions (*p* = 0.025). HR decreased slightly (~ by 5%, *p* = 0.098), and the mean slope of the BRS for up events was increased slightly, but these changes did not reach statistical significance (Table 4). In addition, the TPRI was markedly increased during N2 as compared with baseline values (by ~ 30%, *p* = 0.018) (Table 4). During stable N2 sleep, the VLF component of dBPV significantly increased during the CA segment (25 ± 5% of the 10-min time segments chosen, again and similar to HF patients enrolled corresponding to mild-moderate CA severity) vs. NB (*p* = 0.001), with otherwise neutral effects on other SVB and hemodynamic parameters (Table 4). Similar to our findings in HF patients, data analysis showed that in patients with ICA, central apneas (*n* = 1426; 21 ± 4 s in length) were associated with a ~ 5% decrease in HR (*p* < 0.001), sBP (*p* < 0.001), and dBP (*p* < 0.001) compared with the last 15 s of hyperventilation preceding apnea onset (Fig. 3c).

**Table 4:** Effect of CA on sympathovagal balance parameters and hemodynamic parameters in ICA patients

	Baseline awake (NB)	N2 sleep (NB)	*p* value*	N2 sleep (ICA)	*p* value**
Sympathovagal balance parameters					
HF nuRRI (%)	39.9 ± 29.5	44.2 ± 23.2	*0.07*	41.0 ± 23.2	0.59
LF nuRRI (%)	60.1 ± 29.5	55.8 ± 23.2	*0.07*	59.0 ± 23.2	0.59
LF/HF RRI	3.7 ± 3.3	2.2 ± 2.0	*0.025*	2.3 ± 1.5	0.86
HF nudBPV (%)	22.5 ± 20.6	17.6 ± 15.0	0.51	10.8 ± 15.6	0.13
LF nudBPV (%)	37.6 ± 14.0	43.2 ± 11.5	0.47	30.5 ± 15.3	*0.002*
LF/HF dBPV	2.3(1.5–4.4)	4.1(1.5–6.0)	0.84	5.3(2.7–6.9)	0.88
VLF nudBP (%)	39.9 ± 17.2	39.2 ± 8.7	0.98	62.6 ± 17.9	*0.001*
BRS slope					
Up events (ms/mmHg)	11.5 ± 9.9	16.3 ± 10.9	*0.09*	15.2 ± 11.0	0.77
Down events (ms/mmHg)	14.1 ± 11.0	17.4 ± 8.7	0.62	19.9 ± 14.3	0.52
Hemodynamic parameters					
Heart rate (min^−1^)	62.4 ± 8.8	58.0 ± 6.1	*0.10*	55.9 ± 7.8	0.14
Systolic BP (mmHg)	104.9 ± 11.5	110.4 ± 11.8	0.41	110.2 ± 5.3	0.74
Diastolic BP (mmHg)	63.3 ± 13.0	73.2 ± 9.2	0.12	70.1 ± 8.4	0.19
Stroke volume index (mL/m^2^)	35.0 ± 4.9	31.8 ± 4.2	0.29	36.8 ± 15.1	0.26
Cardiac index (L/min/m^2^)	2.2 ± 0.4	1.8 ± 0.3	0.17	1.8 ± 0.3	0.87
TPRI (dyne s m^2^ cm^−5^)	2997.9 ± 511.7	4002.5 ± 956.3	*0.018*	3842.2 ± 936.8	0.14

Italicized means *p* < 0.05

Values are mean ± standard deviation, median (interquartile range), or number of patients (%)

*For comparison vs. baseline

**For comparison CA vs. NB

*BP*, blood pressure; *BRS slope*, slope of baroreceptor reflex sensitivity (up events and down events); *HF nudBPV*, high frequency component of diastolic blood pressure variability normalized for total power spectra (normalized units); *HF nuRRI*, high frequency component of heart rate variability normalized for total power spectra (normalized units); *ICA*, idiopathic central apnea; *LF nudBPV*, low frequency component of diastolic blood pressure variability normalized for total power spectra (normalized units); *LF/HF dBPV*, relative ratio of low frequency and high frequency component of diastolic blood pressure variability; *LF nuRRI*, low frequency component of heart rate variability normalized for total power spectra (normalized units); *LF/HF RRI*, relative ratio of low frequency and high frequency component of heart rate variability; *NB*, normal breathing; *TPRI*, total peripheral resistance index

## Discussion/conclusion

This is the first study to comparatively investigate the effects of spontaneous CA on SVB and hemodynamics in both HF and ICA patients at night, after adjusting for sleep stage. The most important finding of this study is that the impact of CA on SVB seems to be different depending on the underlying hemodynamic conditions or the presence of HF. In patients with HF, CA had almost neutral effects on SVB compared with NB during N2 sleep. In contrast, in subjects with ICA, CAs were associated with an increase in the VLF component of dBPV. While some oscillatory variations of dBP can be detected within a single cycle of hyperventilation and apnea (either CA in the context of HF, or ICA), when comparing the phases of ventilatory instability to those of NB, an overall neutral hemodynamic effect was observed in both HF patients and heart-healthy subjects.

Transition from wakefulness to sleep was characterized by a decrease in HR and by a shift in HRV (but not BPV) towards vagal predominance over sympathetic control (increased high frequency component, decreased LF, and decreased HF/LF) in heart-healthy subjects, as expected and consistent with a previous study [39]. The same transition was accompanied by a decrease in only HR in patients with HF, while no changes were observed in HRV and BPV, a finding which may be in line with an overall decreased circadian modulation of different physiological signals in HF [3, 6, 9].

It has been hypothesized that CA further increases sympathetic drive in the setting of HF, thus exerting harmful effects if left untreated [4, 40]. Nonetheless, a previous study which applied both polysomnography and right heart catheterization showed that cardiac norepinephrine spillover was mainly related to HF severity rather than to CA severity (based on the AHI) [41]. Moreover, the SERVE-HF trial surprisingly showed that effective suppression of CA during treatment with adaptive servo-ventilation might result in increased mortality [19–21]. This finding gave rise to the hypothesis that CSR may exert compensatory rather than detrimental effects in patients with HF [20, 21]. In particular, Naughton suggested that the hyperventilation phase may enhance stroke volume and attenuate excessive SNA with “physiological features more likely to be compensatory and beneficial than injurious in HF” [22].

We tested this hypothesis by measuring the immediate effects of a CA breathing pattern on SVB and hemodynamics during N2 sleep in HF patients. At least in this cohort of HF patients with only mild to moderate CA, these effects turned out to be neutral; i.e., no significant differences between relevant parameters were found when periods of CA were compared with NB. Patients with HF typically show a CSR pattern, and unstable breathing is characterized by repetitive cycles of hyperventilatory and apneic phases. A possible interpretation of this finding may therefore be that whatever the effect of CA on SVB and cardiac hemodynamics is, this is compensated and counterbalanced during the hyperventilation phase. However, in heart-healthy subjects with normal cardiac output and ICA, an increase in the VLF component was still observed when the same sleep stage was compared with NB.

Three explanations may help explain this finding. Firstly, CA characteristics are different in patients with and without HF (e. g., cycle duration in HF-related CA is 1–2 min vs. 30–45 s in ICA) [5, 24, 25]. Since hypocapnic hyperventilation is likely to attenuate excessive sympathetic drive, longer hyperventilatory phases in HF would explain why the net effect on SVB was neutral in CA due to HF but not in ICA (i.e., due to longer compensatory hyperventilation in HF). Secondly, even similar CAs may result in different autonomic response in patients with and without HF, depending on the background chemoreflex activity and its interaction with other cardiopulmonary reflexes during oscillatory breathing. In particular, HF leads to resetting of several feedback circuits due to forward (hypoperfusion of carotid bodies and kidneys) and backward failure (increased left atrial pressure). Indeed, a previous study from our group pointed towards this theory. Applying simulation of a CSR breathing pattern, it was shown that CSR results in neutral changes effects on SVB in patients with HF [36]. Thirdly, the effects of HF treatment, especially β-blockers, have been previously been shown to dump the firing of sympathetic outflow on peripheral targets (i.e., heart and vessels). Similar effects of β-blocker treatment were obvious in the current study since sympathetic drive was decreased in the awake state in HF compared with ICA patients.

In both patients with HF-related CA and those with ICA, CAs were found to be associated with slight decreases in HR, sBP, and dBP compared with pre-apneic (hyperventilation) levels. These findings support data from physiologic studies of Burnum et al. and a previous study from our group [42, 43], and are also in line with the oscillatory behavior observed in the pulmonary circulation [42]. It is currently unknown whether the net effect of alternating phases of apneas and hyperventilation will always be neutral on sympathovagal balance (as observed in our HF cohort) or also cause a drift in some physiological variables in subjects with different chemoreflex and/or plant gain, circulatory delay, or in different sleep stages. This should be addressed by future studies that should also ideally include some direct measurement of sympathetic outflow, such as cardiac sympathetic nerve recordings in animals or muscle SNA in humans.

Despite its comprehensive approach, this study has, in our opinion, five main limitations. Firstly, previous validation studies using the same non-invasive hemodynamic monitor were only performed in free breathing conditions. Therefore, the results obtained during different breathing maneuvers should be interpreted with caution, especially SVI, CI, and TPRI [27–29]. Nonetheless, even invasive hemodynamic recordings would include uncertainties compared with a steady-state condition, especially when rapid transition from one phase to the other is evaluated (i.e., from hyperventilation to apnea). Secondly, SNA was not measured invasively in this study, i.e., by direct recording of MSNA. Non-invasive recording of HRV and BPV can only provide an indirect measure of SNA. Therefore, it is impossible to understand whether there is a problem of nerve firing or organ response. However, close correlation between MSNA and non-invasive SNA surrogate markers has previously been shown, both in heart-healthy volunteers [44] and HF patients [45], and performance of MSNA during sleep is technically difficult, especially for long recordings. Thirdly, rather short recordings of CA breathing patterns (i.e., 10 min in length) were chosen to assess the effects of CA on SVB. Hence, we cannot exclude that longer periods of CA would increase sympathetic drive in patients with HF. Fourthly, these results are not applicable to different sleep stages and the CA alteration of sleep architecture may also be a cause of HRV/BPV changes during the whole night, as previously described [46]. Finally, our findings may be different in larger populations with more severe CA, different LVEF, background hemodynamics, and feedback set point ideally also measuring MSNA and corrrelating it with the extent of hypocapnia in HF [47]. Hence, neutral findings in the present study must be interpreted again with caution considering the small sample size and pre-selection for the presence of CAs that were “only” present in 20–30% of the 10 minutes of the 10-min time segments evaluated. Taking into account the statistical assumption behind the calculation of the Bonferroni post hoc corrections for multiple *t* tests, actual *p* values reported in Tables 3 and 4 should be interpreted with caution.

In conclusion, CA during sleep is associated with acute hemodynamic effects compared with hyperventilation in both patients with HF and subjects with ICA. When comparing phases of unstable ventilation to those of stable ventilation, no significant change in SVB and hemodynamics was observed in patients with systolic HF, while an increased VLF of dBPV was observed in heart-healthy subjects with ICA. The different effects of CA in different clinical conditions should be thoroughly investigated in future studies to understand if, and in which cases, they may represent a risk and consequently a promising therapeutic target.

## Electronic supplementary material

ESM 1 (DOCX 13 kb).

ESM 2 (DOCX 12 kb).
