# Supplementary material for: Effects of central apneas on sympathovagal balance and hemodynamics at night: impact of underlying systolic heart failure
Source: Sleep Breath. 2020 Jul 22;25(2):965–77. doi: 10.1007/s11325-020-02144-8 (PMC8195752; doi:10.1007/s11325-020-02144-8)
Supplement: Supplementary file 1 — (DOCX 13 kb). [file 11325_2020_2144_MOESM1_ESM.docx]

**Supplemental Table S1: Abbreviations**

AHI apnea-hypopnea index

BPV blood pressure variability

BRS baroreceptor reflex sensitivity

CI cardiac index

DBP diastolic blood pressure

DBPV diastolic blood pressure variability

ECG electrocardiogram

CA central apneas

CSR Cheyne-Stokes respiration

EEG electroencephalogram

HF heart failure

HF high frequency component

*(reflecting parasympathetic drive)*

HR heart rate

HRV heart rate variability

ICA idiopathic central apnea

LF/HF relativ ratio of low frequency component / high frequency component

*A higher LF/HF ratio indicates increased sympathetic drive („stress“)*

LF low frequency component

*(reflecting sympathetic drive and in parts also parasympathetic drive)*

LVEF left ventricular ejection fraction

MSNA muscle sympathetic nerve activity

*(invasive, direct measures of sympathetic drive)*

N2 stage 2 of non-rapid eye movement sleep

NB normal breathing

NT-proBNP N-terminal pro B-type natriuretic peptide

PSG polysomnography

RERA respiratory event-related arousals

RRI RR-Interval (time distance between 2 R-peaks in the ECG)

SBP systolic blood pressure

SNA sympathetic nerve activity

SVB sympathovagal balance

SVI stroke volume index

SVR systemic vascular resistance

TPRI total peripheral resistance index
