# Supplementary material for: Effects of central apneas on sympathovagal balance and hemodynamics at night: impact of underlying systolic heart failure
Source: Sleep Breath. 2020 Jul 22;25(2):965–77. doi: 10.1007/s11325-020-02144-8 (PMC8195752; doi:10.1007/s11325-020-02144-8)
Supplement: Supplementary file 2 — (DOCX 12 kb). [file 11325_2020_2144_MOESM2_ESM.docx]

|  | **Heart failure**  **(n=10)** | **Idiopathic central apnea**  **(n=10)** |
| --- | --- | --- |
| Time in bed of the entire night, h | 7:26±0:46 | 7:18±0:26 |
| Arousal index, /h | 22.9±19.3 | 24.3±25.7 |
| Oxygen desaturation index, /h | 20.6±16.9 | 15.6±19.4 |

**Supplemental Table S2.** Overall Objective sleep characteristics of the study night.
